# Association of a history of gestational diabetes mellitus with osteoporosis, bone mineral density, and trabecular bone score in postmenopausal women

**DOI:** 10.1186/s13098-023-01194-8

**Published:** 2023-10-27

**Authors:** Boqi Lu, Liping Zhang

**Affiliations:** 1 Department of Obstetrics, Haidian District Maternal and Child Health Care Hospital, Beijing, 100080 People’s Republic of China; 2 Department of Obstetrics, Huai’an Maternal and Child Health Care Center, 104 Renmin South Road, Huai’an, 223002 People’s Republic of China

**Keywords:** Gestational diabetes mellitus, Osteoporosis, Bone mineral density, Trabecular bone score, Postmenopausal women

## Abstract

**Background:**

Studies on the association of gestational diabetes mellitus (GDM) with osteoporosis, and bone mineral density (BMD) have been inconsistent. The aim of this study was to investigate the association of a history of GDM with osteoporosis, BMD, and trabecular bone score (TBS) in postmenopausal women.

**Methods:**

Postmenopausal women from the National Health and Nutrition Examination Survey (NHANES) between 2007 and 2010, between 2013 and 2014, and between 2017 and 2018 were retrospectively included in this cross-sectional study. The logistic regression model was used to explore the relationship between GDM and osteoporosis, and a weighted linear regression model was applied to investigate the association between GDM and total femoral BMD, femoral neck BMD, and total TBS. Subgroup analysis of the association between GDM and osteoporosis was performed according to age, body mass index (BMI), and DM (yes or no).

**Results:**

Of the 6732 women included, 253 women (3.76%) had GDM. No significant differences in total femoral BMD, femoral neck BMD, and total TBS were observed between postmenopausal women with and without a history of GDM. However, a history of GDM was associated with a higher risk of osteoporosis in postmenopausal women [odds ratio (OR): 11.18, 95% confidence intervals (CI): 3.64 to 34.27, *P* < 0.001]. There was no significant difference between a history of GDM and osteoporosis in postmenopausal women whom BMI is normal and overweight women. However, there was an association between a history of GDM and osteoporosis in postmenopausal obese women (OR: 26.57, 95% CI 10.23 to 68.98, *P* < 0.001).

**Conclusion:**

A history of GDM was associated with a higher risk of osteoporosis in postmenopausal women, particularly in postmenopausal obese women.

## Background

Osteoporosis is a musculoskeletal disease characterized by loss of bone mass and bone microstructure damage [1]. Postmenopausal women experience a steady loss of bone mass due to aging and hormonal changes, leading to osteoporosis [2]. More than 10 million Americans over the age of 50 years suffer from osteoporosis, and this number is expected to increase in the future with the expected growth of the elderly population [3]. Each year, osteoporosis results in more than 9 million fractures worldwide [4]. Osteoporotic fractures in postmenopausal women are associated with poor quality of life and reduced life expectancy [5]. The gold standard for diagnosing osteoporosis is the measurement of bone mineral density (BMD) using a dual-energy X-ray absorptiometry (DEXA) scan. Given the damage caused by osteoporosis and the fact that BMD is a significant marker of bone mass, it is necessary to identify factors associated with BMD and osteoporosis.

Diabetes mellitus (DM) has been recognized as one of the risk factors for osteoporotic fractures [6]. Gestational DM (GDM), a common complication of pregnancy, is defined as glucose intolerance that first diagnosed during pregnancy [7]. BMD in women with GDM was reported to be lower than those in women with normal blood glucose [8]. Zhu et al. reported that a history of GDM was associated with long-term hip fracture risk and overall fracture risk, but not with calcaneal BMD as measured by ultrasonography [9]. The association between the history of GDM and BMD or osteoporosis requires further investigation. Trabecular bone score (TBS) is a DEXA image-based gray scale structural index that reflects bone microstructure and helps to predict fracture independently of BMD [10]. Previous investigators found significant lower TBS values in DM patients [11, 12]. However, studies on the relationship between DM and TBS have been inconsistent. A study in Korea found that TBS was lower in men with DM than in men without DM; but in women, the observation was not observed [11]. Consequently, a thorough assessment of the relationship between a history of GDM and TBS in postmenopausal women is required.

Herein, the objective of the study was to investigate the association between a history of GDM and osteoporosis, BMD, and TBS in postmenopausal women.

## Methods

### Study design and participants

This study was a cross-sectional study. The data were obtained from a cross-sectional survey-the National Health and Nutrition Examination Survey (NHANES), a large, comprehensive, and regularly updated sample of the non-hospitalized U.S. population [13]. Every 2 years, the National Health and Nutrition Examination Survey (NHANES) gathers information to assess the health and nutritional status of Americans. The database included information on the research population’s demographics, physical examination, laboratory test results, nutrition, and questionnaires [13]. This study collected data from 2007 to 2010, 2013 to 2014, and 2017 to 2018. Inclusion criteria were: (1) postmenopausal women; (2) those with the assessment of BMD; (3) those who had a history of GDM assessed in the NHANES database. Participants with missing information on key covariates were excluded. The study was performed in accordance with the Declaration of Helsinki, and approved by the National Center for Health Statistics (NCHS) Ethics Review Board (ERB) (Continuation of Protocol #2005-06; continuation of Protocol #2005-06; continuation of Protocol #2011-17; Protocol Code #2018-01, effective beginning October 26, 2017; continuation of Protocol Code #2011-17, effective through 26 October 2017).Thus, the requirement of ethical approval for this was waived by the Institutional Review Board of Huai’an maternal and child health care center. Written informed consent was not required as this study was based on publicly available data. All methods were performed in accordance with the relevant guidelines and regulations.

### Data collection

Data were collected including age (years), race/ethnicity, educational level, marital status, poverty income ratio (PIR), alcohol and smoking status, physical activity, hypertension, DM, previous fracture, parental fracture, glucocorticoid use, anti-osteoporosis therapy, body mass index (BMI), cords-25-hydroxyvitamin D2 (25OHD2) + 25OHD3 (nmol/L), energy/day (kcal), serum vitamin D (mcg), osteoporosis, total femoral BMD (gm/cm^2^), femoral neck BMD (gm/cm^2^), TBS (bone degeneration, no bone degeneration, and unknown).

Race/ethnicity was categorized as Mexican American, other Hispanic, non-Hispanic White, non-Hispanic Black, and other races including multiracial. Educational level was identified as less than 9th grade, 9th–11th grade, high school grade/general educational development (GED) or equivalent, some college or AA degree, and college graduate or above. Smoking status was categorized into yes and no based on the following questions: Have you smoked at least 100 cigarettes in your entire life?” and “Do you now smoke cigarettes?”. Alcohol consumption identification was based on answers to questions regarding having had at least 12 drinks in the past year (ALQ101) or drinking more than 12 drinks in a lifetime (ALQ110). Using the formula BMI = weight (kg)/height (m)^2^ to determine BMI. Physical activity was evaluated by determining the MET (Met*min). Alcohol status, smoking status, hypertension, DM, previous fracture, parental fracture, glucocorticoid use, and anti-osteoporosis therapy were classifies as yes versus no.

### Measurements and outcomes

A history of GDM was identified using the questions “During your pregnancy, were you ever told by a doctor, or other health professional that you had DM, sugar DM, or GDM?”, and “How old were you when you were first told you had DM during a pregnancy?”. Women who answered yes to the question were considered to have a history of GDM. Women were identified as developing DM if they reported having a diagnosis of DM (other than during pregnancy) or, or if they had a haemoglobin (A1C) level was ≥ 6.5%, a fasting plasma glucose level ≥ 126 mg/dL, or 2-h plasma glucose level ≥ 200 mg/dL if they had not previously been diagnosed with DM [13].

The primary outcome of the study was osteoporosis. The secondary outcomes were total femoral BMD, femoral neck BMD, and total TBS. Osteoporosis was determined based on total femoral BMD (gm/cm^2^) < 0.64 or femoral neck BMD (gm/cm^2^) < 0.56. Total femoral BMD and femoral neck BMD were measured using a dual energy X-ray absorptiometry (DEXA) scan.

### Statistical analysis

Continuous data were described by mean and standard error Mean (S.E), and the comparisons between groups were made by weighted T-test (R package “survey”: “svyttest”). Enumeration data were described in terms of case size and component ratio (N(%)), and the χ^2^ test was used for comparison between groups (R package “survey”: “svychisq”). Data cleaning, data processing, and statistical analyses were performed using SAS 9.4 (SAS Institute, Cary, NC, USA), and R version 4.2.0 (2022-04-22 ucrt).

For the dichotomous outcomes of osteoporosis, weighted univariate and multivariate logistic regression models were used to explore the association between GDM and osteoporosis. For total femoral BMD, femoral neck BMD, and total TBS, weighted univariate linear regression and weighted multivariate linear regression models were applied to investigate the association between GDM and total femoral BMD, femoral neck BMD, and total TBS. For the outcome of osteoporosis, the multiple logistic regression model adjusted for age, education, marital status, PIR, alcohol status, previous fracture, parental fracture, and BMI; for total femoral BMD, age, race, education, marital status, PIR, hypertension, DM, previous fracture, parental fracture, glucocorticoid use, anti-osteoporosis therapy, BMI, serum vitamin D, and energy were adjusted for; for femoral neck BMD, age, race, marital status, previous fracture, parental fracture, anti-osteoporosis therapy, and BMI were adjusted for; for total TBS, age, race, education, PIR, alcohol status, hypertension, DM, previous fracture, and vitamin D were adjusted for. Subgroup analyses were conducted to further examine the association between GDM and osteoporosis. The evaluation indexes were odds ratio (OR), and 95% confidence interval (CI). Alpha = 0.05, a *P* < 0.05 was considered statistically significant.

## Results

### Baseline characteristics of included participants

**Fig. 1:**
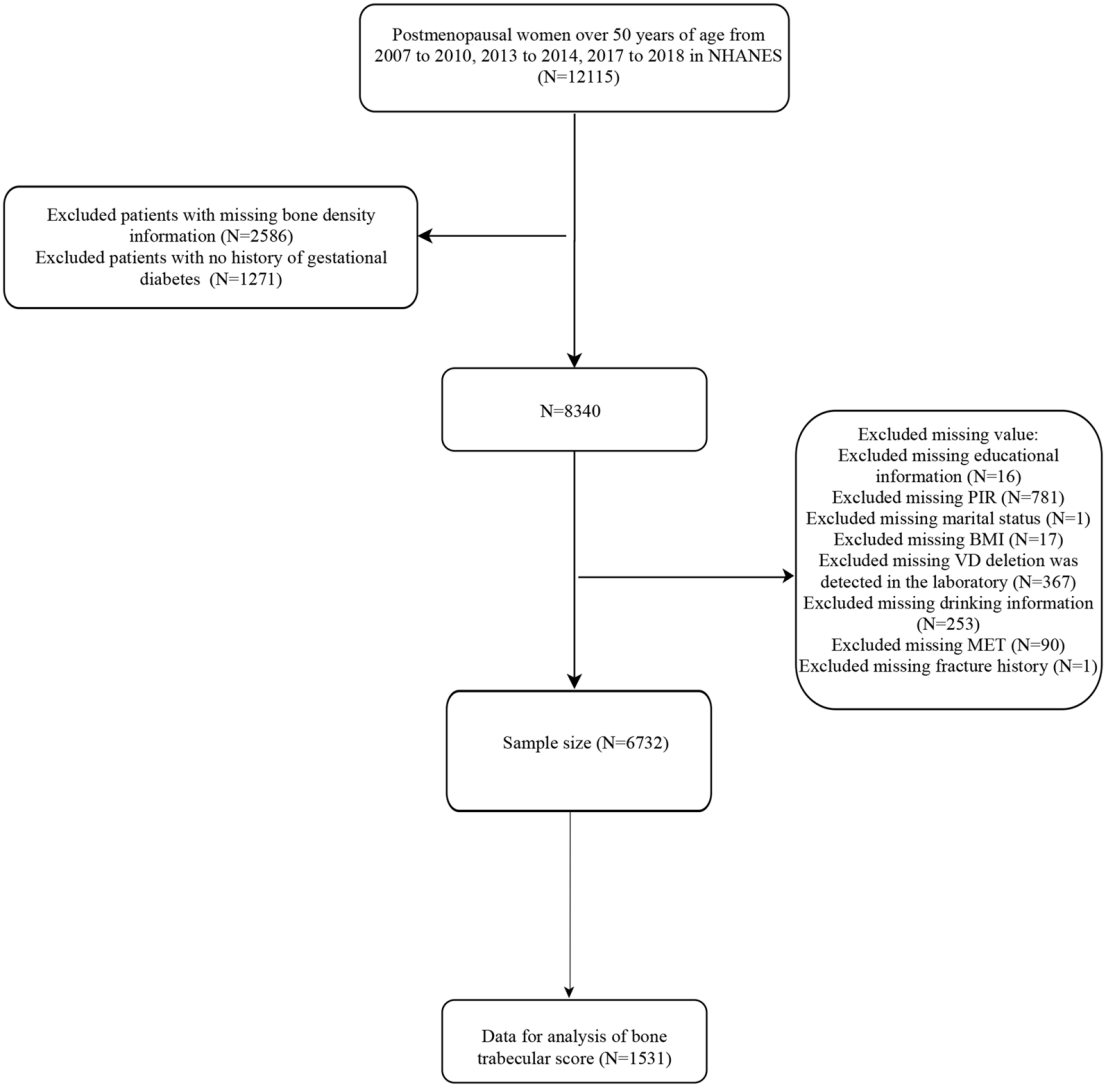
The diagram flow of the data collection

Based on the inclusion and exclusion criteria, 6732 women were included in this study. The flow chart of the data collection is shown in Fig. 1. Of the 6732 women, 253 women (3.76%) were with GDM. The mean age of the included women was 65.76 (0.28) years, with the majority of women (82.99%) being of non-Hispanic White race/ethnicity. Significant differences in age, education level, smoking status, DM, BMI, and osteoporosis were observed between women with GDM and those without GDM (*P* > 0.05 for each). Table 1 shows the characteristics of the study participants.

**Table 1:** Basic characteristics of the study participants

Variables	Total (n = 6732)	GDM		Statistics	*P*
		Yes (n = 253)	No (n = 6479)		
Age, years, Mean (S.E)	65.76 (0.28)	62.65 (1.51)	65.89 (0.29)	t = − 2.11	
Race/ethnicity, n (%)				χ^2^ = 0.506	0.973
Mexican American	653 (3.02)	36 (3.79)	617 (2.98)		
Other Hispanic	598 (3.15)	28 (3.93)	570 (3.12)		
Non-Hispanic White	4052 (82.99)	121 (80.80)	3931 (83.08)		
Non-Hispanic Black	923 (5.81)	37 (6.04)	886 (5.80)		
Other race-including multi-racial	506 (5.03)	31 (5.43)	475 (5.01)		
Education level, n (%)				χ^2^ = 21.365	< 0.001
Less than 9th grade	469 (2.81)	17 (2.26)	452 (2.83)		
9–11th grade	791 (8.76)	12 (3.07)	779 (9.00)		
High school grade/GED or equivalent	1737 (25.93)	37 (10.08)	1700 (26.58)		
Some college or AA degree	2195 (33.90)	140 (61.35)	2055 (32.77)		
College graduate or above	1540 (28.60)	47 (23.24)	1493 (28.82)		
Marital status, n (%)				χ^2^ = 0.146	0.702
Married	3436 (59.11)	149 (54.19)	3287 (59.32)		
Other (widowed, divorced, separated, never married, living with partner)	3296 (40.89)	104 (45.81)	3192 (40.68)		
Ratio of family income to poverty, Mean	3.33 (0.08)	3.84 (0.31)	3.31 (0.07)	t = 1.77	0.082
Alcohol status, n (%)				χ^2^ = 2.190	0.139
Yes	2626 (29.60)	79 (19.26)	2547 (30.03)		
No	4106 (70.40)	174 (80.74)	3932 (69.97)		
Smoked at least 100 cigarettes in life				χ^2^ = 2.823	0.093
Yes	2659 (39.09)	87 (25.41)	2572 (39.65)		
No	4073 (60.91)	166 (74.59)	3907 (60.35)		
MET, Met*min, Mean (S.E)	579.38 (67.29)	292.11 (87.14)	591.23 (70.17)	t = − 2.63	0.011
Hypertension, n (%)				χ^2^ = 2.674	0.102
No	1645 (28.95)	49 (16.57)	1596 (29.46)		
Yes	5087 (71.05)	204 (83.43)	4883 (70.54)		
DM, n (%)				χ^2^ = 29.385	< 0.001
Yes	5247 (81.96)	101 (39.11)	5146 (83.73)		
No	1485 (18.04)	152 (60.89)	1333 (16.27)		
Broken or fractured a hip, n (%)				χ^2^ = 1.701	0.192
Yes	182 (2.52)	6 (7.52)	176 (2.31)		
No	6550 (97.48)	247 (92.48)	6303 (97.69)		
Parental fracture, n (%)				χ^2^ = 1.205	0.272
No	5892 (84.55)	225 (71.94)	5667 (85.07)		
Yes	840 (15.45)	28 (28.06)	812 (14.93)		
Glucocorticoid use, n (%)				χ^2^ = 3.723	0.054
No	6076 (89.48)	219 (69.93)	5857 (90.29)		
Yes	656 (10.52)	34 (30.07)	622 (9.71)		
Anti-osteoporosis therapy, n (%)				χ^2^ = 1.925	0.165
No	5696 (84.66)	212 (67.69)	5484 (85.36)		
Yes	1036 (15.34)	41 (32.31)	995 (14.64)		
BMI, kg/m^2^, Mean (S.E)	28.49 (0.25)	33.37 (2.20)	28.29 (0.23)	t = 2.31	0.024
25OHD2 + 25OHD3, nmol/L, Mean (S.E)	90.85 (1.75)	83.71 (4.16)	91.14 (1.74)	t = − 1.99	0.051
Calcium, mg, Mean (S.E)	1693.75 (29.74)	1887.31 (154.33)	1685.76 (30.14)	t = 1.30	0.198
Energy, kcal, Mean (S.E)	3383.99 (49.15)	3765.67 (193.91)	3368.24 (50.89)	t = 1.97	0.053
Vitamin D, mcg, Mean (S.E)	8.46 (0.23)	9.25 (0.82)	8.43 (0.24)	t = 0.93	0.354
Osteoporosis, n (%)				χ^2^ = 7.472	0.006
No	5888 (87.62)	218 (62.45)	5670 (88.66)		
Yes	844 (12.38)	35 (37.55)	809 (11.34)		
Total femoral BMD, gm/cm^2^, Mean (S.E)	0.84 (0.00)	0.85 (0.03)	0.84 (0.00)	t = 0.34	0.737
Femoral neck BMD, gm/cm^2^, Mean (S.E)	0.70 (0.01)	0.60 (0.10)	0.71 (0.00)	t = − 1.10	0.276
TBS, n (%)				χ^2^ = 9.074	0.011
No bone degeneration	791 (11.09)	17 (2.74)	774 (11.43)		
Bone degeneration	740 (7.54)	6 (2.65)	734 (7.74)		
Unknown	5201 (81.37)	230 (94.61)	4971 (80.83)		

GDM: gestational diabetes mellitus; GED: general educational development; DM: diabetes mellitus; BMI: body mass index; BMD: bone mineral density; T: weighted T test; χ^2^: χ^2 ^test

### Associations of GDM with osteoporosis, total femoral BMD, femoral neck BMD, and total TBS

The result of the unadjusted model showed that women with a history of GDM had a higher risk of osteoporosis (OR: 4.82, 95% CI 1.35 to 17.21, *P* = 0.018). From the model adjusting for variables, a history of GDM was associated with postmenopausal osteoporosis (OR: 11.18, 95% CI 3.64 to 34.27, *P* < 0.001). The association between GDM and osteoporosis is presented in Table 2. However, no associations between GDM and total femoral BMD (β: − 0.04, 95% CI − 0.08 to 0.01, *P* = 0.079), femoral neck BMD (β: − 0.13, 95% CI − 0.29 to 0.03, *P* = 0.118), and total TBS (β: − 0.04, 95% CI − 0.17 to 0.10, *P* = 0.588) were observed.

**Table 2:** Associations of GDM with osteoporosis, total femoral BMD, femoral neck BMD, and total TBS

Outcomes	Variables	Model 1		Model 2	
		OR/β (95% CI)	*P*	OR (95% CI)	*P*
Osteoporosis	GDM (Yes)	4.82 (1.35–17.21)	0.018	11.18 (3.64–34.27)	< 0.001
Total femoral BMD	GDM (Yes)	0.01 (− 0.04–0.06)	0.348	− 0.04 (− 0.08–0.01)	0.079
Femoral neck BMD	GDM (Yes)	− 0.11 (− 0.32–0.09)	0.273	− 0.13 (− 0.29–0.03)	0.118
Total TBS	GDM (Yes)	− 0.08 (− 0.26–0.09)	0.362	− 0.04 (− 0.17–0.10)	0.588

Model 1 is an unadjusted model; for the outcome of osteoporosis, model 2 adjusted for age, education, marital status, PIR, alcohol status, previous fracture, parental fracture, and BMI; for total femoral BMD, age, race, education, marital status, PIR, hypertension, diabetes, previous fracture, parental fracture, glucocorticoid use, anti-osteoporosis therapy, BMI, serum vitamin D, and energy were adjusted for; for femoral neck BMD, age, race, marital status, previous fracture, parental fracture, anti-osteoporosis therapy, and BMI were adjusted for; for total TBS, age, race, education, PIR, alcohol status, hypertension, DM, previous fracture, and vitamin D were adjusted for

*GDM* gestational diabetes mellitus, *BMD* bone mineral density, *TBS* trabecular bone score, *OR* odds ratio, *CI* confidence interval

### Subgroup analysis of associations between GDM and osteoporosis

The result of the subgroup analysis indicated that a history of GDM was related to osteoporosis in postmenopausal women aged ≥ 65 years (OR: 14.21, 95% CI 2.64 to 76.35, *P* = 0.003) or aged < 65 years (OR: 5.16, 95% CI 1.07 to 24.8, *P* = 0.046). There was no significant difference between a history of GDM and osteoporosis among postmenopausal women with normal weight (OR: 8.12, 95% CI 0.53 to 123.41, *P* = 0.138) and overweight (OR: 0.32, 95% CI 0.05 to 2.13, *P* = 0.245). However, among postmenopausal women with obesity, an association between a history of GDM and osteoporosis was observed (OR: 26.57, 95% CI 10.23 to 68.98, *P* < 0.001). A history of GDM was associated with postmenopausal osteoporosis, regardless of whether the women had DM (OR: 13.92, 95% CI 3-63.58, *P* < 0.001) had not (OR: 7.42, 95% CI 1–89.17, *P* = 0.006). The subgroup analysis of associations between GDM and osteoporosis is shown in Table 3.

**Table 3:** Subgroup analysis of associations between GDM and osteoporosis

Subgroups	OR (95% CI)	*P*
Age		
≥ 65	14.21 (2.64–76.35)	0.003
< 65	5.16 (1.07–24.82)	0.046
BMI		
Normal	8.12 (0.53–123.41)	0.138
Overweight	0.32 (0.05–2.13)	0.245
Obesity	26.57 (10.23–68.98)	< 0.001
DM		
Yes	13.92 (3.62–53.58)	< 0.001
No	7.42 (1.89–29.17)	0.006

Age, education, marriage, PIR, alcohol status, previous fracture, parental fracture, and BMI were adjusted for

*GDM* gestational diabetes mellitus, *CI* confidence interval, *OR* odds ratio, *DM* diabetes mellitus, *BMI* body mass index

## Discussion

Postmenopausal women are considered to be at high risk of developing osteoporosis [14], so evaluation of factors related to BMD, TBS, osteoporosis may help to develop preventive strategies for DM-related bone changes. We found no significant difference in total femoral BMD, femoral neck BMD, and total TBS between postmenopausal women with and without a history of GDM, but, a history of GDM was associated with a higher risk of osteoporosis among postmenopausal women. There was no significant difference between a history of GDM and osteoporosis in postmenopausal women with a normal weight and overweight women. However, there was an association between a history of GDM and osteoporosis in postmenopausal obese women.

Both DM and osteoporosis are increasing health problems worldwide [15]. Having type 2 DM (T2DM) has been reported as a risk factor for osteoporosis and osteoporotic fractures [15]. Wang et al. demonstrated that poor glycemic control was related to lower levels of bone formation, which may increase the risk of bone fracture in postmenopausal women with T2DM [16]. Karimifar et al. found that osteopenia and osteoporosis are more common in diabetic postmenopausal women compared to non-diabetic postmenopausal women [17]. In this study, a history of GDM was associated with higher osteoporosis risk in postmenopausal women. Regarding the mechanisms between a history of GDM and postmenopausal osteoporosis, several data suggest an effect of advanced glycosylation end products (AGEs) on collagen and bone cells. AGEs accumulating in collagen have been shown to stimulate IL-6 production in human bone cells [18], inhibit osteoblast phenotypic expression, differentiation, and mineralization, inhibit type 1 collagen synthesis, and promote the formation of weak bridges between collagen fibers, resulting in the reduced bone strength and increased osteoclast resorption [19, 20]. Alterations in IGF-1 levels have also been associated with bone abnormalities. IGF-I is also synthesized by osteoblasts and is a regulator of bone cell metabolism [21]. Several studies have shown reduced IGF-1 activity when glucose and AGE levels are high, suggesting osteoblastic resistance to the effects of IGF-1 [22, 23]. Additionally, chronic inflammation may also link the bone abnormalities in DM [24]. Inflammation induced by obesity inhibits the synthesis and secretion of adiponectin from adipose tissue, which may have consequences on bone metabolism [25]. We did not find an association between GDM and total femoral BMD, femoral neck BMD, and total TBS. However, a previous study by Ma et al. reported that the BMD levels in the GDM group were lower than in the normal group [26]. The association between GDM and BMD of the total femoral, BMD of the femoral neck, and total TBS needs to be further investigated.

There was no association between a history of GDM and osteoporosis in postmenopausal normal and overweight women, but there was an association between a history of GDM and osteoporosis in postmenopausal obese women. Previous studies have suggested a positive association between BMI and BMD [27, 28]. A Mexican study of postmenopausal women with normal weight, overweight or varying degrees of obesity found a positive and significant association between BMI and lumbar, total hip, and femoral neck BMD [28]. Obesity has effects on a number of hormones known to act on bone, and so may act on bone through endocrine pathways. Adipocytes express aromatase as the main source of estrogen in postmenopausal women [29]. High fat mass is associated with higher circulating estradiol, so aromatase activity is likely to contribute to the beneficial effects of fat on bone, particularly in postmenopausal women. Pancreatic and gut hormone secretion is altered in obesity and may influence bone metabolism. Insulin, amylin, and pepsin are increased in obesity and may have direct effects on bone cells to increase bone formation and decrease resorption. Insulin may also have indirect beneficial effects on bone by decreasing hepatic sex hormone-binding globulin production, which increases the bioavailability of estrogen and androgens [30]. Promoting a healthy lifestyle and reducing BMI in women with GDM may be justified to improve bone health and prevent future osteoporosis.

Based on the population-representative database of NHANES, this study explored the association between the history of GDM and BMD, TBS, and the risk of osteoporosis in postmenopausal women, which filled the gap in related fields to a certain extent. Understanding the factors associated with BMD and osteoporosis risk in postmenopausal women with a history of GDM has important implications for medical plans and individual patients. Nonetheless, the study does have its limitations. First, the history of GDM in this study was obtained by a self-reported doctor-diagnosed GDM, which inevitably has a certain recall bias. Second, the cross-sectional nature of the survey limits our ability to identify aetiological factors. Third, the detection data of TBS in the database are limited, and the proportion of GDM in the population with TBS is extremely low. Therefore, prospective and large-sample studies are needed to further investigate the effect of a history of GDM on TBS.

## Conclusions

A history of GDM was associated with a higher risk of osteoporosis in postmenopausal women, and there was an association between a history of GDM and osteoporosis in postmenopausal women with an obese BMI.

## Data Availability

The datasets generated and/or analyzed during the current study are available in the NHANES database,https://wwwn.cdc.gov/nchs/nhanes/.

## Abbreviations

BMD: Bone mineral density
DEXA: Dual-energy X-ray absorptiometry
GDM: Gestational DM
TBS: Trabecular bone score
NHANES: National Health and Nutrition Examination Survey
PIR: Poverty-to-income ratio
BMI: Body mass index
GED: General educational development
